# Restrictive versus liberal oxygenation targets in patients with acute heart failure and pulmonary congestion–A protocol for a Randomized Controlled Trial (The REDOX-AHF trial)

**DOI:** 10.1371/journal.pone.0349791

**Published:** 2026-05-22

**Authors:** Ida Arentz Taraldsen, Anne Sophie Overgaard Olesen, Jasmin Dam Lukoschewitz, Ejvind Frausing Hansen, Olav Wendelboe Nielsen, Christian Hassager, Jens Jakob Thune, Maryam Noory, Arzu Karamat, Jens Dahlgaard Hove, Johannes Grand

**Affiliations:** 1 Department of Cardiology, Copenhagen University Hospital - Amager and Hvidovre‌‌, Hvidovre, Denmark; 2 Faculty of Health and Medical Sciences, University of Copenhagen, Copenhagen‌‌, Denmark; 3 Department of Cardiology, Copenhagen University Hospital – Frederiksberg and Bispebjerg, Copenhagen, Denmark; 4 Department of Respiratory Medicine‌‌, Copenhagen University Hospital - Amager and Hvidovre, Hvidovre, Denmark; 5 O2matic ApS, Herlev, Denmark; 6 Novo Nordisk, Bagsværd, Denmark; 7 Department of Cardiology, Copenhagen University Hospital – Rigshospitalet, Copenhagen‌‌, Denmark; RWJBH: RWJBarnabas Health, UNITED STATES OF AMERICA

## Abstract

**Introduction:**

Acute heart failure (AHF) is the cause of one in twenty hospital admissions. Supplemental oxygen therapy is a routine treatment in the management of patients with dyspnea, including those with AHF. Current guidelines recommend oxygen therapy as part of the initial treatment, if peripheral oxygen saturation (SpO_2_) is < 90% or partial pressure of arterial oxygen (PaO_2_) is < 60 mmHg to correct hypoxemia. There is no clear evidence favoring restrictive or liberal oxygenation strategies in patients with acute heart failure. The aim of this study is to compare restrictive and liberal oxygenation strategies in patients hospitalized for acute heart failure.

**Methods:**

The study is an investigator-initiated, multicenter, prospective, double-blinded, randomized clinical trial. Patients are randomized in a 1:1 fashion to receive one of the two treatment-strategies: liberal oxygenation (SpO_2_ target of 96%) or restrictive oxygenation (SpO_2_ target of 90%). The intervention is administrated double-blinded using an automated oxygen administration device, titrating the oxygen supplementation towards the intended target SpO_2_ without it being visible for patients, investigators or clinical staff. The primary outcome is pulmonary congestion, measured by remote dielectric sensing (ReDS) after 24 hours, adjusted for baseline measurements.

**Conclusion:**

This study will explore the current uncertainty regarding restrictive and liberal oxygenation targets in acute heart failure.

## Introduction

Acute heart failure (AHF) is a common cause of hospitalization, particularly among older adults, and is associated with high morbidity and mortality [1,2]. With an ageing population, the burden of AHF is expected to increase further [3]. Patients with AHF may present with hypoxemia caused by pulmonary congestion, pleural effusion or concomitant pulmonary comorbidities such as pneumonia and chronic obstructive pulmonary disease (COPD), all of which impair the lungs’ capacity to oxygenate the blood [4]. In combination with reduced cardiac output, this may compromise end-organ oxygen delivery [4].

Current guidelines recommend supplemental oxygen therapy from the pre-hospital phase and upon admission for patients with peripheral oxygen saturation (SpO_2_) below 90% or at higher values if the treating physician finds it clinically indicated [4]. However, there are currently no recommendations regarding a target SpO_2_ level in patients with acute heart failure.

Patients with AHF are often treated with oxygen supplementation to maintain an SpO_2_ above 95%, according to treatment algorithms and triage systems, such as the National Early Warning score (NEWS) [5]. Oxygen exerts various vasoactive effects throughout the vascular system [6,7]. In the coronary arteries and myocardium, it acts as a vasoconstrictor [6], and several small physiologic studies have suggested deleterious effects of high oxygen levels on cardiac function, including reduced cardiac output and increased systemic‌‌ vascular resistance [6,8–10]. Conversely, in the pulmonary system, oxygen acts as a vasodilator, decreasing pulmonary vascular resistance [7]. In theory, this might be beneficial in conditions with increased right ventricular afterload [11].

Despite oxygen being one of the first interventions initiated in the acute treatment phase of AHF, the optimal oxygen saturation target and treatment strategy remain unclear. To improve patient outcome, it is crucial to find the right balance between harmful effects of hypo- and hyperoxemia. We will compare liberal and restrictive oxygenation strategies for patients hospitalized with AHF. We hypothesize that restrictive oxygenation is associated with improved decongestion, compared to liberal oxygenation.

## Methods

### Study design and intervention

This is an investigator-initiated, prospective, double-blinded, multi-center, randomized clinical trial. The trial is registered at ClinicalTrials.gov (ID: NCT05613218). After informed consent and completion of screening procedures, patients are randomized in a 1:1 fashion to receive one of two treatment-strategies:

- Liberal oxygenation group with an SpO_2_ > 95% (targeting 95–97% - with no additional oxygen given when above 97%).
- Restrictive oxygenation group with an SpO_2_ > 89% (targeting 89–91% - with no additional oxygen given when above 91%).

The restrictive oxygenation target is based on the recommendation to give oxygen when SpO_2_ is < 90% in the current guidelines [4]. The liberal oxygenation target is based on current usual care, following the NEWS treatment algorithm, where it is recommended to keep SpO_2_ above 95%.

**Fig 1:**
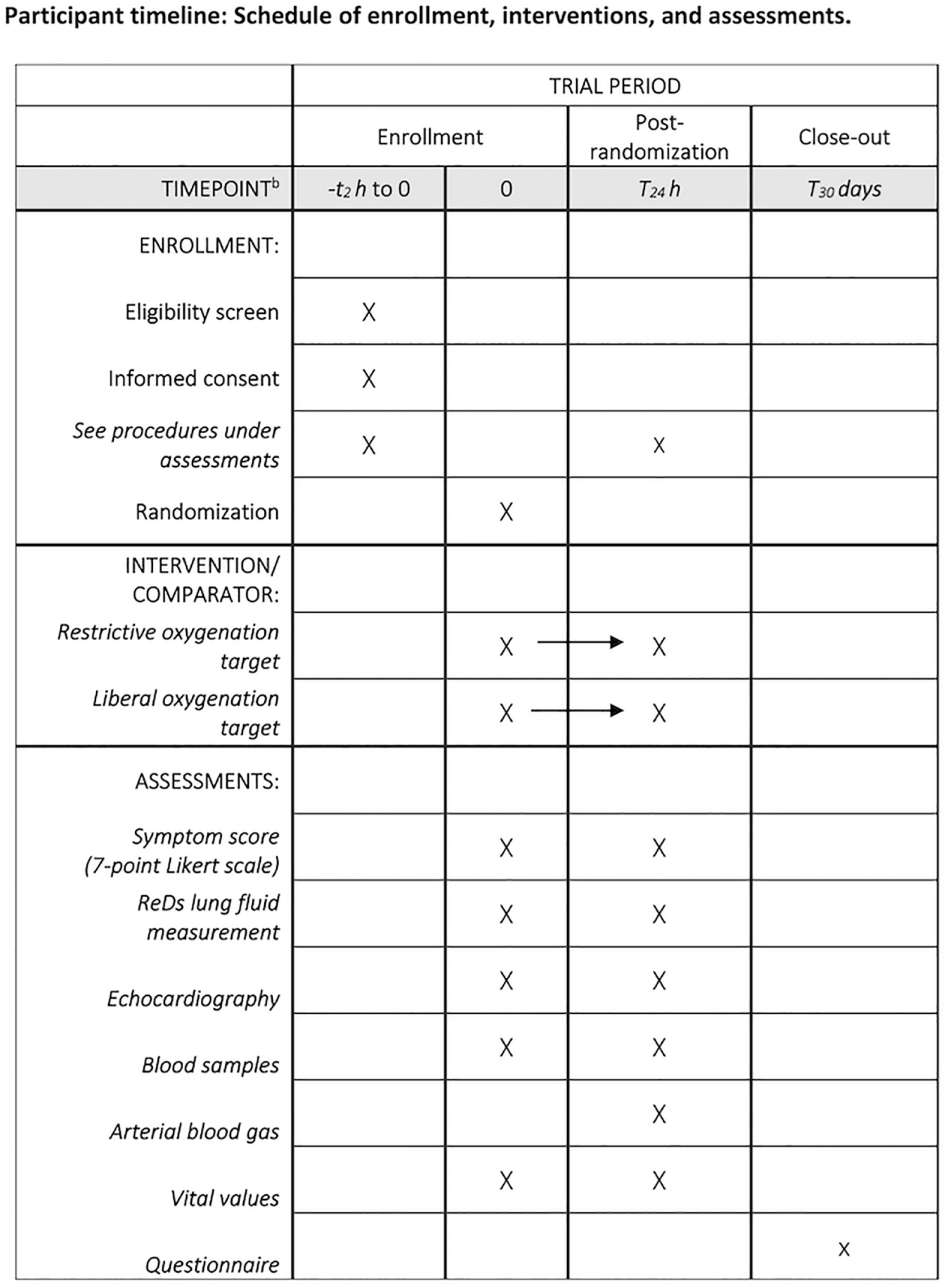
SPIRIT Participant timeline. Schedule of enrollment, interventions, and assessments.

Trial investigators will screen the sites for eligible patients and ask for written informed consent. As soon as possible hereafter, screening procedures and randomization will be performed. This will typically happen in the emergency department (ED), but patients may be included up to 4 hours after admission to the cardiac ward. Randomization will be performed using the web-based system REDCap (REDCap Consortium, Nashville, USA) hosted at Capital Region Denmark. See Fig 1 for the SPIRIT schedule of enrollment, interventions, and assessments and S1 File for the Spirit 2025 Check list.

The intervention will be administered and blinded by an automated oxygen administration device O2matic (O2matic Ltd., Herlev, Denmark), which has previously been demonstrated to be superior to nurse administered oxygen administration, to keep saturation within a target saturation interval [12,13]. The device enables investigators to select one of two prespecified settings, including two different target saturation intervals (89–91% and 95–97%), without being unblinded. During the 24 h intervention, O2matic will titrate the patient’s oxygen supplementation every second using continuous SpO_2_-measurements obtained via a pulse oximeter placed on the finger or an ear. O2matic can deliver an oxygen flow of 0–15 L/min, enabling titration of oxygen to the two target saturations without oxygen flow or SpO_2_ being visible to the patients, investigators or treating personnel. The 24-hour time frame was selected, as previous studies demonstrate that most patients hospitalized due to AHF are no longer on oxygen supplementation after 24 hours from admission and as a pragmatic compromise between clinical relevance and feasibility, as some patients may remain oxygen-dependent beyond this time point [14]. Health personnel are allowed to measure SpO_2_ during the intervention, as a part of the usual monitoring of the patient but will not be able to check how much oxygen the patient is receiving. If there is an audible increase in oxygen demand, personnel can as appropriate change from a standard nasal cannula to a humidifier, mixed with 5 L/min atmospheric air, or a facial mask.

### Setting and patient population‌‌

All patients are included in-hospital by trial investigators. We started inclusion in March 2024 and expect to complete inclusion within two years. The trial is terminated after the last patient has been assessed after 30 days. The study is performed at two sites in Denmark:

Bispebjerg Hospital, Bispebjerg Bakke 23, 2400 Copenhagen
Amager Hvidovre Hospital, Kettegård Alle 30, 2650 Hvidovre

We include patients with acute heart failure, based on a clinical diagnosis, based on symptoms and clinical or radiological signs of congestion. A complete list of inclusion and exclusion criteria are listed in Table 1.

**Table 1:** Inclusion and exclusion criteria.

Inclusion criteria	Exclusion criteria
1. Age ≥ 18 years	1. More than 4 hours from admission to the cardiac ward to randomization
2. Acute (within minutes to days) onset or worsening of subjective dyspnea	2. Suspected infection or sepsis
3. Oxygen saturation ≤ 92% or need of oxygen	3. Known severe COPD with FEV1 < 50%
4. At least one of the following (clinical or radiological signs of congestion): a. Pulmonary rales b. Chest X-ray or CT with pulmonary congestion c. Lung ultrasound with multiple B-lines	4. Systolic blood pressure < 90 mmHg

CT, computed tomography; COPD, chronic obstructive pulmonary disease; FEV1, forced expiratory volume in 1 second; mmHg, millimeter mercury.

### Study examinations

Prior to randomization, all included patients will undergo echocardiography, focused lung ultrasound (FLUS) and Remote Dielectric Sensing (ReDS). After 24 hours, the intervention will be discontinued, and oxygen will be withheld for 10 minutes before repeating the study assessments, which will then also include an arterial blood gas analysis. If the patient severely desaturates, with SpO_2_ levels < 85%, the arterial blood gas and ReDS measurement will be performed as quickly as possible, and the patient will receive supplemental oxygen during the echocardiography.

ReDS is performed by applying a clip over the patients’ right shoulder with a posterior and anterior sensor, with a thin layer of clothing in between. In one minute, the lung fluid content is estimated, calculated by the average dielectric coefficient of the thorax tissue. ReDS is an FDA-approved, wearable device that allows quick and accurate lung fluid measurements, noninvasively. The lung fluid content is measured within the mid-region of the right lung and presented as the percentage of the lung volume composed of fluid. Normal values for non-congested people is 20%−35% of fluid content [15]. ReDS has been demonstrated to have consistent reproducibility with pulmonary congestion on computed tomography (CT) and elevated measurements have been associated with higher risk of readmission [16,17].

The FLUS is performed with an abdominal C1-6 transducer or a cardiac M5Sc-D transducer if the abdominal transducer is unavailable. We will perform the FLUS according to the standardized 14-zone protocol, as described by Laursen et al [18]. The patient will first be positioned in a supine position with the bed elevated 45 degrees for the anterior and lateral zones, thereafter the patient will be in a seated position for the posterior zones. If the patient is unable to mobilize to seated position, a modified protocol including the 8 anterior and lateral projections will be performed.

The baseline TTE will be performed bedside in a supine position, while the 24-hour TTE may be performed in the recumbent position, if the patient’s clinical condition is stabilized sufficiently. The TTE will primarily be performed with a Vivid E95 (General Electric, Norway) ultrasonic system with a 4Vc transducer, in cases where this is unavailable, the examination will be performed on a Vivid S70N with a M5Sc-D transducer. See S2 File. Supplemental methods: TTE for a detailed description of the TTE procedure.

### Outcome measures

#### Outcome measures.

The primary outcome is change in pulmonary parenchymal fluid content (%) measured with the ReDS technology (Sensible Medical, Netanya, Israel), from baseline to 24-hour, preceded by 10 minutes without oxygen supplementation.

Secondary outcomes include arterial blood gas oxygen concentration after 24 hours preceded by 10 minutes without oxygen supplementation, patient-reported dyspnea on a 7-point Likert scale after 24 hours, from marked worsening to marked improvement, change in respiratory rate from baseline to 24 hours and time to freedom from oxygen-supplementation. See Table 2 for exploratory outcomes.

**Table 2:** Exploratory outcomes.

	Outcome measurements
1	Change from baseline in biomarkers (N-Terminal Pro-Brain Natriuretic Peptide (NT-proBNP), troponin T and creatinine, to 24 hours from admission.
2	Days alive and out-of-hospital to day 30
3	Days alive out-of-ICU until day 30.
4	All-cause mortality at day 30.
5	Length of in-hospital stay
6	Multiple B-lines in at least two areas bilaterally on lung ultrasound identifying interstitial syndrome (yes/no).
7	Change in total B-line count from baseline to 24 hours.
8	SpO_2_, FiO_2_, Blood pressure and heart rate after 24 hours.
9	Arterial blood gas oxygen concentration of lactate, pCO_2_ and pO_2_ after 24 hours preceded by 10 minutes without oxygen supplementation.
10	Quality of life and depression: EQ-5D-5L after 30 days.
11	Number of serious adverse events

ICU, intensive care unit; FiO2, fraction of inspired oxygen; pCO2, partial pressure of carbon dioxide; pO2, partial pressure of oxygen.

#### Feasibility outcomes.

To evaluate the success of the double-blinded oxygen administration we have the following feasibility outcomes: Time within targeted SpO_2_-interval (89–91% in restrictive group, 95–97% in liberal group), overall difference in SpO_2_ using a repeated measurements mixed model, number of patients where the intervention is terminated (opt out) before 6 hours and time with severe hypoxia (defined as SpO_2_ < 85%).

### Predefined sub-studies

#### Echocardiographic sub-study.

A predefined sub-study will investigate the changes in echocardiographic parameters, defined as changes from baseline to 24 hours. The primary outcome is E/e’, where e’ is calculated as the average of the lateral and septal measurements in the 4CH view.

Secondary outcomes are listed in Table 3. The sub-study will also evaluate a subgroup identified with elevated estimated pulmonary pressure.

**Table 3:** Secondary outcomes for the echocardiographic sub-study.

	Outcome measurements
1	Tricuspid regurgitation gradient (TR-gradient).
2	Estimated pulmonary pressure, assessed by the inferior vena cava, including its respiratory variation and the TR-gradient.
3	LVEF (auto-EF, eyeballing and Simpsons biplane).
4	Stroke volume derived from the velocity time integral of the left ventricular outflow tract.
5	GLS (global longitudinal strain (%)), calculated as the mean from 4CH, 2CH and 3CH view
6	Average s’, e’ and a’ (averaged from septal, lateral, inferior, anterior, posterior and anteroseptal mitral annular positions in tissue doppler images).
7	Left atrial volume indexed (LAVi)
8	Time to peak strain for each left ventricular wall segment during systole.
9	Tricuspid annular plane systolic excursion (TAPSE), measured with M-mode.

4CH, 4-chamber view; LVEF, left ventricular ejection fraction; E, mitral inflow E-wave.

#### Bayesian sub-study.

The effects of the intervention on pre-defined outcomes will be analyzed using Bayesian statistical methods. The primary estimand will be the posterior mean difference between groups in change in ReDS at 24 hours. Results will be reported as posterior means with 95% credible intervals, along with the posterior probability that the restrictive oxygenation strategy reduces lung fluid content compared with the liberal strategy. As evidence supporting specific oxygenation strategies in patients with suspected acute heart failure is limited, weakly informative priors will be used. The exact model specification, including likelihood functions, prior distributions, and any additional analyses, will be prespecified in a separate statistical analysis plan finalized prior to data analysis.

### Sample size calculation

A previous study by Amir et al validated the ReDS technology, using chest computed tomography as golden standard, in 15 patients with acute decompensated heart failure (ADHF) and 16 patients without ADHF. They found a mean fluid content of 39.8 + 6.8% in patients with ADHF, with comparable results when estimating fluid content by chest computed tomography [15]. As the minimal clinically important difference in lung fluid content, as measured by ReDS has not been established, we chose 4% as a pragmatic effect size, intended to reflect a potentially meaningful reduction in pulmonary congestion.

To find a 4% change in lung fluid content (from 39.8% to 35.8%) from baseline to 24-hours in an intention-to-treat analysis, we need to include 61 patients in each group. This is calculated with a standard deviation of 6.8 and a power of 90% and a α-level of 5%. We expect minimal dropouts and crossovers.

We will include patients over an estimated period of two years in two centers. We will include patients until we have reached 122 patients without any missing values for the primary outcome, to ensure that we can conclude on our primary hypothesis.

Enrollment was completed in February 2026, after initial submission of this protocol.

### Ethical considerations

The study is approved by the local ethics committee (H-22062699) and is conducted in accordance with national legislation on medical research involving humans. See S3 File. For the Protocol approved by ethical committee. Patients will give written consent before enrollment and participation will not interfere with diagnostics and treatment. Patients will be given oxygen to levels within current recommendations and monitored as usual by health personnel. If a substantial increase in oxygen demand is observed, health personnel will contact the treating physician, who will evaluate if further stabilizing measus1

es are needed, as per standard clinical practice. The treating physician is encouraged to consult the investigator before discontinuing the intervention, if this is deemed clinically appropriate.

### Statistical analysis plan

Baseline characteristics will be reported as mean ±SD if normally distributed, and as median (25^th^ percentile-75^th^ percentile) if non-normally distributed and compared with T-test or Wilcoxon rank-sum test as appropriate. Categorical variables will be presented as numbers (percentages) and will be compared with Fisher’s exact test. The primary exposure variable is defined as allocation to the intervention group, compared to the control group. Repeated measurements mixed models will be made to analyze continuous variables with multiple measurements. These models will only be applied when we have sufficient data.

The primary endpoint will be analyzed with an analysis of covariance (ANCOVA) model, with lung fluid estimate after 24 hours as outcome measure and baseline lung fluid estimate as a covariate.

Survival data will be graphically presented with Kaplan-Meier curves for each group and compared with log-rank test. Multivariable Cox proportional hazard models will be applied to assess differences in time to death between treatment groups. These models will sequentially be adjusted for the interaction between treatment allocation, and each of the predefined covariates. Further, models stratified by individual components of the predefined covariates will be made as hypothesis generating.

Binary outcomes will be evaluated using univariable and multivariable logistic regression models after examining assumptions of linearity and proportionality. Hazard ratios (HRs) and Odds ratios (ORs) with corresponding two-sided 95% confidence intervals (CI) will be presented. Continuous outcomes will be evaluated using univariable and multivariable linear regression models and will be logarithmic transformed to approximate normal distribution as appropriate.

Multivariable models are adjusted for: *age, sex and body mass index.*

The covariables are pre specified before analyses and were chosen based on a consensus of the steering committee based on experiences from previous trials of AHF. Interaction between covariables will be tested by adding the interaction term to the models. The variates will be categorized, if they are not normally distributed.

For all the prespecified covariables (*Admission SBP, age, sex, body mass index, LVEF at admission (>40, <=40), NT-proBNP, eGFR, supraventricular arrhythmia at admission, comorbidities (history of heart failure, obstructive pulmonary disease)*, we will perform subgroup analyses and investigate interaction with treatment effect with a forest plot.

All analyses will be conducted in the modified intention-to-treat (ITT) (all randomized patients fulfilling the inclusion criteria and excluding those, which consent have been withdrawn) population. As a secondary analysis, we will conduct the analysis for the primary and secondary endpoints in an as-treated population (defined as the SpO_2_-target the patient was kept within for a majority of the intervention-period, accounting for potential cross-over), as well as in a per-protocol analysis.

As a sensitivity analysis we will analyze the primary and secondary outcomes in the subgroup of patients with elevated pro-BNP (defined as ≥ 53 pmol/L for patients aged < 50 years, ≥ 106 pmol/L for patients aged 50–74 years and ≥ 212 pmol/L for patients aged ≥ 75 years, using the highest measured value. For patients with supraventricular tachycardia, we will use more conservative cut offs: ≥ 159 pmol/L for patients aged < 50 years, ≥ 318 pmol/L for patients aged 50–74 years and ≥ 636 pmol/L for patients aged ≥ 75 years). For the primary outcome we will also do a separate sensitivity analysis adjusting for PaO_2_ to investigate whether any difference is caused by a difference in pulmonary blood volume, due to different degrees of pulmonary vasodilation [19,20].

Data extraction and data analyses will be performed before unblinding, except for the data from the O2matic device, as this is not possible without being unblinded. P-values less than 0.05 will be considered statistically significant. Statistical analyses are performed using the SAS statistical software, version 9.4 (SAS Institute, Cary, NC) and in R version 3.3.3 (R Foundation for Statistical Computing, Vienna, Austria).

## Discussion

Oxygen therapy is one of the most frequently used treatments in patients with AHF, yet oxygen saturation levels in this population range widely. About half of patients present with hypoxemia (PaO₂ < 60 mmHg), but notably, a large proportion of normoxemic patients (SpO₂ ≥ 90%) also receive supplemental oxygen upon admission [21,22]. To our knowledge, there is only one small randomized controlled trial investigating optimal oxygen targets in AHF, and management of normoxemic patients therefore remains largely empirical [4,23,24].

SpO_2_ is used as a non-invasive surrogate marker for PaO_2_ which can directly be measured by a blood gas. Hypoxemia is defined as PaO_2_ < 60 mmHg, corresponding to SpO_2_ < 90%. In our study we titrate the oxygen supplementation by SpO_2_, even though PaO_2_ is the real target. There might be a discrepancy between the two measurements, that we cannot account for, especially in the liberal oxygenation group.

**Fig 2:**
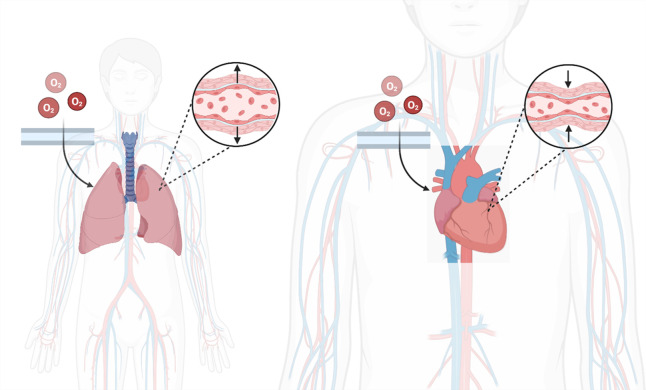
Physiologic effects of oxygen.

There are several large systematic reviews on oxygen targets in broader populations, such as acutely ill patients, which have found neutral or harmful effects of liberal oxygen therapy on mortality and serious adverse events [25,26]. This may not apply to patients with AHF, due to the different vasoactive effects throughout the cardiopulmonary system (Fig 2) [6,9–11]. High levels of oxygen have been demonstrated to reduce cardiac output, increase the systemic vascular resistance, reduce coronary blood flow and reduce myocardial oxygen consumption [9,6]. On the other hand, it has also been shown to decrease mean pulmonary artery pressure, in patients with pulmonary hypertension [7]. How these different physiologic effects interact together in AHF is uncertain.

Oxygen therapy has been investigated in other cardiovascular conditions, especially in acute myocardial infarction, where the routine use of oxygen therapy, compared to air, was not found to be beneficial on all-cause mortality in several systematic reviews [27,28]. In a more selective group with ST-elevation myocardial infarction, Alves et al found a signal towards a decrease in short-term mortality in the group with high oxygen supply. However, the relative risk for recurring myocardial infarction was lower in the low oxygen group.

Our primary outcome is change in pulmonary parenchymal fluid content measured with ReDS which gives an unbiased measurement of pulmonary congestion, compared to other methods such as lung ultrasound, where the risk of inter- and intraobserver variability is larger [29,30]. A limitation is that the sample size is not powered to conclude on clinical outcomes, such as mortality. Due to our pragmatic inclusion and exclusion criteria, there is also an innate risk of a mixed population, where some patients’ primary cause of admission is not acute heart failure. This might weaken the power of our analysis, but it is also a strength, as it well mimics clinical practice, where patients are treated according to the current tentative diagnosis, even though this for some patients might change after few hours to days, as more clinical and paraclinical‌‌ examinations are performed.

With this trial, we aim to provide reliable evidence regarding restrictive vs liberal oxygenation targets in patients with AHF. Given the lack of randomized clinical trials directly addressing oxygen strategies in AHF, this trial will shed light on an unmet need – investigating the physiological and clinically relevant potential harmful effects of liberal versus restrictive oxygenation strategies and guide future treatment recommendations. We also hope to give new insight on the methodological aspects, guiding the design of larger future trials, including identification of optimal saturation thresholds and patient subgroups most likely to benefit from specific oxygen strategies. The next step should be larger, sufficiently powered randomized controlled trials evaluating clinical outcomes.

## Supporting information

S1 File Spirit 2025 check list. (DOCX)

S2 File Supplemental methods: TTE [31]. (DOCX)

S3 File Protocol approved by ethical committee. (DOCX)

S1 Fig Graphical abstract‌‌. (TIFF)
