# Supplementary material for: Restrictive versus liberal oxygenation targets in patients with acute heart failure and pulmonary congestion–A protocol for a Randomized Controlled Trial (The REDOX-AHF trial)
Source: PLoS One. 2026 May 22;21(5):e0349791. doi: 10.1371/journal.pone.0349791 (PMC13196945; doi:10.1371/journal.pone.0349791)
Supplement: S2 File — (DOCX) [file pone.0349791.s002.docx]

**Supplementary**

**Supplemental methods: TTE**

Three consecutive cardiac cycles are recorded for each view. A full examination is performed according to the guidelines from the American Society of Echocardiography, including 2D, Doppler and M-mode [32]. To the best of the patient’s ability, depending on the degree of respiratory distress, all diastolic Doppler measurements are obtained at end-expiration. Tissue Doppler images (TDI) are obtained in both apical 4 chamber (CH), 2CH and 3Ch view as well as of the right ventricle. Pulsed-wave-TDI is obtained from the lateral and septal mitral annulus in the 4CH view and at the lateral tricuspid annulus.
