# Supplementary material for: Restrictive versus liberal oxygenation targets in patients with acute heart failure and pulmonary congestion–A protocol for a Randomized Controlled Trial (The REDOX-AHF trial)
Source: PLoS One. 2026 May 22;21(5):e0349791. doi: 10.1371/journal.pone.0349791 (PMC13196945; doi:10.1371/journal.pone.0349791)
Supplement: S3 File — (DOCX) [file pone.0349791.s003.docx]

STUDY PROTOCOL

REstrictive versus liberal OXygenation targets in patients with Acute Heart Failure and pulmonary congestion – a randomized clinical pilot-trial

Acronym: The REDOX-AHF trial

Version: 2.0

Sponsor Protocol Code number: REDOX-1

Datatilsynet, En-Suite nummer: P-2022-770

Ethical committee number: H-22062699

ClinicalTrials.gov number: NCT05613218

Primary Investigator:

Johannes Grand, MD PhD

Copenhagen University Hospital Amager-Hvidovre

Department of Cardiology

Kettegård Alle 30, 2650 Hvidovre Copenhagen

Denmark

# Trial synopsis

Title: RESTRICTIVE VERSUS LIBERAL OXYGENATION TARGETS IN PATIENTS WITH ACUTE HEART FAILURE AND PULMONARY CONGESTION – A RANDOMIZED CLINICAL PILOT-TRIAL

Background:

One million hospitalizations occur each year with a primary diagnosis of acute heart failure in the USA, with comparable numbers in Europe. Most patients with acute heart failure are treated with supplemental oxygen during hospitalization and guidelines recommend initiation of oxygen therapy if SpO2 <90% (class I, level C). However, no clinical trials of oxygen targets in humans with acute heart failure investigating clinically relevant endpoints have been performed.

Primary objective:

To investigate the effect of a restrictive vs. liberal oxygenation-strategy in patients hospitalized with acute heart failure.

Hypothesis:

Restrictive oxygenation is associated with improved clinical outcome compared to liberal oxygenation.

Design: Investigator-initiated, prospective, randomized, blinded, multi-center, controlled trial.

Intervention:

Patients will be randomized 1:1 in the emergency department to either liberal or restrictive oxygenation after providing informed written consent.

(1) Liberal oxygenation group = SpO2 target of 96%.

(2) Restrictive oxygenation group = SpO2 target of 90%.

Patients will have nasal cannula or oxygen mask placed as the usual care, and oxygen is titrated to the prespecified target range.

Consented patients will be randomly allocated to study groups via the automated web-based system within REDCap. The allocation will be concealed. Time at randomization will be considered as study time zero (T0). All patients will receive usual standard of care except for their oxygen-administration.

The intervention-phase will be 24 h, and hereafter oxygen therapy will be at the discretion of the treating physician.

Inclusion criteria:

1. Age ≥ 18 years
2. Acute (within minutes to days) onset or worsening of subjective dyspnea
3. Oxygen saturation <92% (on arterial blood gas) or need of oxygen
4. At least one of the following clinical or radiological signs of congestion:
   - - 1. Pulmonary rales
       2. Chest X-ray or CT with pulmonary congestion
       3. Lung ultrasound with multiple B-lines

Exclusion criteria:

1. More than 4 hours from hospital admission to randomization
2. Suspected infection or sepsis
3. Known severe pulmonary disease
4. Systolic blood pressure <90 mmHg

Primary outcome:

Pulmonary parenchymal fluid content after 24 hours preceded by 10 minutes without oxygen-supplementation.

Secondary outcomes:

1. Arterial blood gas concentration after 24 hours preceded by 10 minutes without oxygen-supplementation.
2. All-cause mortality at day 30
3. Days alive out-of-hospital to day 30
4. Time to freedom from oxygen-supplementation

Sample Size/Study Duration:

To have 90 % power to find a difference of 4 % in lung fluid content (change from 39.8% to 35.8%) at 24 hours with a standard deviation of 6.8, we need to include **122 patients (61 in each group).** The α-level is 0.05, and the beta is 10%. We expect minimal dropouts. The study will include patients over an estimated period of 1 years in two centers.

#

**TABLE OF CONTENTS**

[Trial synopsis 2](#_Toc116815237)

[1.Trial data 7](#_Toc116815238)

[1.1 Steering Group 7](#_Toc116815239)

[1.2 Trial sites: 7](#_Toc116815240)

[1.3 Participating Sites 7](#_Toc116815241)

[1.4 Official Approvals 7](#_Toc116815242)

[1.5 Data Safety Monitoring Board 7](#_Toc116815243)

[2. Background 8](#_Toc116815244)

[3. Trial objectives and hypotheses 11](#_Toc116815245)

[4. Study design 11](#_Toc116815246)

[5. Intervention 11](#_Toc116815247)

[5.1 Detailed intervention and suggested initial treatment 11](#_Toc116815248)

[6. Setting and patient population 12](#_Toc116815249)

[6.1 Setting 12](#_Toc116815250)

[6.2 study sites 13](#_Toc116815251)

[6.3 Inclusion criteria 13](#_Toc116815252)

[6.4 Exclusion criteria 13](#_Toc116815253)

[6.5 Estimated Study start and duration 13](#_Toc116815254)

[7. Outcomes 13](#_Toc116815255)

[7.1 Primary outcome 14](#_Toc116815256)

[Pulmonary parenchymal fluid content measured by ReDS after 24 hours preceded by 10 minutes without oxygen-supplementation. 14](#_Toc116815257)

[7.2 Secondary outcomes 14](#_Toc116815258)

[8. Sample size calculations and statistical analysis plan 14](#_Toc116815259)

[8.1 Expected enrollment 15](#_Toc116815260)

[9. Pre-defined study-procedures 15](#_Toc116815261)

[10. ETHICAL CONSIDERATIONS 16](#_Toc116815262)

[10.1 ETHICAL JUSTIFICATION 16](#_Toc116815263)

[9.2 Informed consent 17](#_Toc116815264)

[11. Monitoring 18](#_Toc116815265)

[11.1 Good Clinical Practice monitoring 18](#_Toc116815266)

[11.2 Access to Source Data and Documentation 18](#_Toc116815267)

[12. Statistical analyses plan 18](#_Toc116815268)

[12. Data collection and management 20](#_Toc116815269)

[11.1 Data sources 20](#_Toc116815270)

[11.2 Data collection process 20](#_Toc116815271)

[13. Clinical treatment outside the treatment allocation 24](#_Toc116815272)

[14. ADVERSE EVENT REPORTING 24](#_Toc116815273)

[14.1 Definitions 25](#_Toc116815274)

[14.1.1 Adverse events 25](#_Toc116815275)

[14.1.2 Serious adverse events 26](#_Toc116815276)

[13.5 Reporting 27](#_Toc116815277)

[13.6 Reporting Procedures for All Adverse Events 27](#_Toc116815278)

[14. Timeline and enrollment 28](#_Toc116815279)

[15. Funding 28](#_Toc116815280)

[16. Publication plan 29](#_Toc116815281)

[16.1 Design paper 29](#_Toc116815282)

[16.2 Main results paper 29](#_Toc116815283)

[16.3 Auxiliary manuscripts / post-hoc analyses 30](#_Toc116815284)

[17. Data sharing 30](#_Toc116815285)

[18. Tasks and responsibilities 30](#_Toc116815286)

[18.1 Sponsor/PI 30](#_Toc116815287)

[18.2 Steering group 30](#_Toc116815288)

[19. References 31](#_Toc116815289)

[Appendix 1: Steering group conflicts of interest 34](#_Toc116815290)

[Appendix 2: Generic flow chart containing information on when and how to include patients 35](#_Toc116815291)

[Appendix 4: study-related procedures 36](#_Toc116815292)

[Appendix 5: Timeline 38](#_Toc116815293)

[Appendix 6: Acronyms and abbreviations 39](#_Toc116815294)

PREFACE

The study ‘REDOX-AHF-trial’ will be conducted in accordance with this protocol. The trial protocol follows the International Conference on Harmonization (ICH) Guidelines [1], and the Standard Protocol Items: Recommendations for Interventional Trials (SPIRIT) statement [2]. The primary investigator and steering committee wrote this protocol. Any substantial changes or amendments will be documented and communicated to relevant parties.

# 1.Trial data

Sponsor and Primary investigator Johannes Grand, MD PhD

## 1.1 Steering Group

Ida Arentz Taraldsen [ida.arentz.taraldsen.01@regionh.dk](mailto:ida.arentz.taraldsen.01@regionh.dk)

Jens Dahlgaard Hove [Jens.Dahlgaard.Hove@regionh.dk](mailto:Jens.Dahlgaard.Hove@regionh.dk)

Ejvind Frausing Hansen, ejvind.frausing.hansen@regionh.dk

Olav Wendelboe Nielsen, [olav.wendelboe.nielsen@regionh.dk](mailto:olav.wendelboe.nielsen@regionh.dk)

Christian Hassager [Christian.Hassager@regionh.dk](mailto:Christian.Hassager@regionh.dk)olav.wendelboe.nielsen@regionh.dk

Johannes Grand, [Johannes.grand@regionh.dk](mailto:Johannes.grand@regionh.dk)

*Conflicts of interest: The members of the steering committee have no conflicts of interest related to the current trial. A list of all conflict of interests is provided in Appendix 1.*

## 1.2 Trial sites:

Coordinating site:

Amager-Hvidovre Hospital, Copenhagen, Denmark

## 1.3 Participating Sites

Hospital: Site investigator:

DK Bispebjerg Hospital, Bispebjerg Bakke 23, 2400 København Olav W. Nielsen

DK Amager Hvidovre Hospital, Kettegård Alle 30, 2650 Hvidovre Jens Hove

## 1.4 Official Approvals

Datatilsynet, En-Suite nummer: P-2022-770Ethical committee number: H-22062699

ClinicalTrials.gov number: NCT05613218

## 1.5 Data Safety Monitoring Board

No DSMB will be needed for this trial.

# 2. Background

Acute heart failure is a leading cause of hospitalizations in patients aged >65 years and over one million emergency department hospitalizations occur each year with a primary diagnosis of acute heart failure in the USA, with comparable numbers in Europe [3, 4]. In USA, heart failure is the most expensive reason for admission and re-admission for older patients [5]. With increasing age this burden will likely continue to rise [6]. Up to a third of patients hospitalized with acute heart failure will die or be re-hospitalized within 3 months [7–10]. The continued poor outcome for acute heart failure is in contrast to the progress made in other cardiovascular fields, such as chronic heart failure and acute coronary syndrome [11].

Heart failure is a chronic syndrome characterized by symptoms and clinical findings such as dyspnea, pulmonary and systemic congestion [12]. The syndrome is a consequence of myocardial dysfunction or structural cardiac disease that may lead to either of or a mixture of forward and backward failures [12]. An acute heart failure-episode is seen when heart failure present itself de novo with abrupt symptoms leading to an emergency department visit or when patients with known heart failure have an acute exacerbation requiring hospitalizing [13]. Acute heart failure refers to rapid onset of symptoms and clinical signs of heart failure leading to an unplanned hospital admission or an emergency department visit [12]. Patients with acute heart failure should be evaluated immediately and subsequent treatment administered in the acute setting within the first few minutes to hours [12].

Four main clinical presentations are emphasized in 2021 European guidelines for heart failure. The four phenotypes can be separated based on the presence of signs of congestion (no congestion = dry; congestion = wet) and/or peripheral hypoperfusion (normoperfusion = warm; hypoperfusion = cold) and may require different treatments [12]. Acutely decompensated heart failure is the most common form of acute heart failure, accounting for 50-70% of presentations [14]. Acute pulmonary oedema occurs in 13-25 % of presentations [15]. <5 % presents with isolated right ventricular failure or cardiogenic shock [14, 15]. The division into phenotypes is mostly academic and guidelines emphasize that overlaps between them should be considered. Besides isolated RV-failure, pulmonary congestion with or without systemic congestion is a main characteristic of acute heart failure and is present in above 50% of cases.

Treatment of pulmonary congestion

Despite the critical importance of initial management, data are sparse regarding the impact of early treatment delivered in the emergency department [13]. Current guidelines recommend three therapies to be commenced: First, oxygen and/or continuous positive airway pressure / non-invasive positive-pressure-ventilation. Second, i.v. diuretics, and third, i.v. vasodilators may be considered when SBP is >110 mmHg (previous guidelines had 90 mmHg as cutoff [16]), to reduce LV afterload.

Lung fluid content

The ability to quantify the degree of pulmonary congestion in AHF can help guide treatment and the pulmonary water content can be assessed noninvasively by the remote dielectric sensing (ReDS) device (Sensible Medical, Netanya, Israel). ReDS is an FDA-approved, wearable device developed by Sensible Medical Innovations that allows clinicians and other health care providers to quickly and accurately measure lung fluid in patients, noninvasively. The lung fluid content is measured within the mid-region of the right lung. The measurement is presented as the percentage of the lung volume composed of fluid, (normal values for non-congested people is 20%-35% of fluid content) [25]. This novel noninvasive electromagnetic energy-based technology [26] is intended to quantify changes in lung fluid content. The device measures the dielectric properties of tissues. Low power electromagnetic signals are emitted across the thorax through the lung and the characteristics of the signals received after passing through the tissue are related to their dielectric properties which, in turn, are mostly determined by the lung's fluid content. The dive is superior to bio-impedance-based devices which are affected by many factors in addition to lung fluid content (e.g., electrode placement, body habitus, fat content, skin moisture) and doesn’t have the need for electromagnetic radiation associated with chest CT. In a validation study of acute heart failure patients, fluid content quantified by ReDS™ was highly correlated to chest CT [26].

Oxygenation

Supplemental oxygen therapy has been used for more than a century and is a routine treatment in the management of patients with dyspnea in the emergency department, including those with acute heart failure (AHF) [17]. Even in absence of the arterial hypoxemia, oxygen is often administered in these patients on the basis of the clinicians’ or patients’ belief that it will improve organ oxygenation [17]. Current guidelines recommend the initiation of oxygen therapy (class 1,c) as part of initial treatment [12] if SpO2 <90% or PaO2 <60 mmHg to correct hypoxemia. However, there is a remarkable absence of clinical evidence in this area of treatment [12]. While there is consensus among guideline-writers regarding the treatment of severe hypoxaemia (low O2 saturation levels or SpO2), it is unclear whether oxygen should be administered in absence of severe hypoxemia.

Oxygen is a pulmonary vasodilator, and higher oxygen concentrations is associated with lower pulmonary vascular resistance. This may theoretically unload a failing right ventricle, which can be an advantage in cases of increased RV-afterload, such as acute pulmonary embolism. Contrary, it is believed that hyperoxemia should be avoided, as it causes systemic vasoconstriction including brain and myocardium, and a reduction in cardiac output in high doses [18]. Several physiologic studies have suggested deleterious effects of hyperoxia on cardiac function [19]. In general oxygen is considered a systemic arterial vasoconstrictor and associated with reduced coronary blood flow and myocardial oxygen consumption [19, 20]. In 10 patients with congestive heart failure Haque et al. showed a reduction in cardiac output, stroke volume and increase in pulmonary capillary wedge pressure and systemic vascular resistance when administrated 100% oxygen on a face mask [20]. Mak et al. gave 100% oxygen to two groups of patients with and without heart failure and found hyperoxia associated with increased LV filling pressures in both patient-groups irrespective of heart failure. The authors concluded, that caution should be used in the administration of inspired oxygen to normoxic patients, especially in the setting of heart failure [21]. ESC 2021 guidelines for heart failure recommend oxygen therapy to be initiated first of all treatments in hypoxemic AHF. Yet guidelines are cautious regarding recommendations on the use of supplemental O2 therapy in non-hypoxaemic patients with AHF. The guidelines cite just one study by Park et al. of 13 patients with chronic heart failure, where oxygen administration was shown to cause vasoconstriction and a reduction in cardiac output [18]. Of note, no clinical studies of oxygen in acute heart failure are cited by contemporary guidelines and yet oxygen is recommended if SpO2 <90% or PaO2 <60 mmHg [12]. In a recent large trial of resuscitated cardiac arrest patients, a low oxygenation target did not result in different outcome compared with a higher oxygenation target [22]. In a large trial of oxygen therapy in suspected acute myocardial infarction, Hofmann et al. found no benefit of routine use of supplemental oxygen [23].

In a pilot-trial by Sepehrvand et al., 25 patients with AHF were allocated to high (≥96%) and 25 patients to low (90–92%) SpO2 targets [24]. They found no differences in key primary or secondary endpoints, which the trial was underpowered for, but saw a significant prolonged hospital length-of-stay in the low target group (p=0.01) [24]. One limitation to that trial, beside the small sample size, was the time from admission to inclusion in the trial of median 11.4 (IQR 7.3, 13.5) hours.

Several therapeutic goals for patients admitted with pulmonary congestion are pertinent. Since patients suffer from severe dyspnea, a rapid relieve should be one therapeutic goal. Additionally, since a more targeted emergency treatment will potentially reduce the need for mechanical ventilation and intensive care admissions, another goal is to decrease hospital length of stay. Still, such achievements should transpire without patients experiencing organ hypoxemia or worsening heart failure in the days after the initial treatment. Adverse reactions to the treatment, such as hypoxemia or hypercapnia, should be balanced with the potential benefit in the emergency setting. Previous studies of acute heart failure have included patients up to and above 11 hours post-admission, why trial-based evidence to guide treatment in the first hours of admission is non-existing.

The effects of oxygen administration for acute heart failure in the emergency setting are still unknown and a clinical equipoise exist between restrictive or liberal oxygenation. The different strategies should be investigated in prospective trials to optimize acute treatment.

# 3. Trial objectives and hypotheses

To investigate the effect of a restrictive vs. liberal oxygenation-strategy on outcomes in patients hospitalized with AHF.

Hypothesis:

Restrictive oxygenation is associated with improved outcome compared to liberal oxygenation.

# 4. Study design

The study is an investigator-initiated, prospective, randomized, blinded, multi-center, controlled trial. Following successful completion of screening procedures, patients will be randomized in a 1:1 fashion to receive either of the 2 treatments-strategies.

# 5. Intervention

Random allocation to 1 of 2 strategies:

- Liberal oxygenation group = SpO2 target of 96%.
- Restrictive oxygenation group = SpO2 target of 90%.

5.1 Detailed intervention and suggested initial treatment

Immediately after admission, the standard of care-treatment can be initiated as per discretion of the treating physician. Oxygen can be administered freely. The attending physician at the site will assess the patient’s eligibility for inclusion in the trial. After successful screening, the patient will be asked for written informed consent from study investigators. The sites will aim to obtain the consents as soon as possible. Patients will have nasal cannula or oxygen mask placed as the usual standard of care.

Patients will be screened and randomized in the emergency department to either liberal or restrictive oxygenation after providing informed written consent and oxygen is titrated to the prespecified target range using an automated feedback device (O2MATIC). Consented patients will be randomly allocated to study groups via the web-based system within REDCap. The allocation will be concealed. Time at randomization will be considered as study time zero (T0). All patients will receive usual standard of care except for their O2 management. The intervention will last for 24 h. After 24 h, patients will be switched over to usual care for oxygen therapy. We selected the 24 h time frame because in previous studies most patients with AHF were no longer on oxygen after 24 h [27]. If the treating physician thinks, that the patient need another oxygenation target and it is thought to be harmful to continue with the allocated target, the intervention-phase can be stopped prematurely.

Blinding

The intervention will be administrated double-blinded using the O2MATIC-device, which will titrate the oxygen-supplementation towards the intended target SpO2 without it being visible for patients, investigators or treating personnel. The robot will be programmed to deliver the intervention, record minute-by-minute data of oxygen-supplementation and SpO2. Furthermore, the robot will give an alarm if SpO2 drops below 88%. During the intervention-phase, SpO2 will not be measured by the nurses.

#### Titration of oxygen using an automated feed-back device (O2matic) for patients with acute heart

#### failure needing oxygen-therapy

A Danish research group has developed a closed-loop system for oxygen-administration, O2matic, which is developed at Hvidovre Hospital, Denmark [28]. The first prototypes have been tested at the Copenhagen Academy of Medical Education and Simulation (CAMES), where it has been found in simulation models of COPD exacerbations that O2matic is better than fixed oxygen dosage to keep the oxygen saturation within the desired range. O2matic developed and manufactured in a version that is in accordance with the requirements of the Medical Device Directive product has been CE marked. It has also been tested in a recorded and controlled clinical study in which automated and continuous oxygen monitoring was assessed versus manual control in patients with inpatient exacerbation of COPD.

# 6. Setting and patient population

## 6.1 Setting

The trial includes patients in the acute emergency setting. All patients are included in-hospital in the associated emergency departments. Patients are recruited from participating centers by the attending cardiologist or emergency physician. The first patient-contact is passed on from clinical staff to the research group, as the research group prior to obtaining consent do not have access to patient records. The first contact is made via the clinical staff, so that a potential trial participant has the option to decline further information about the project before contact are made from the research group. From trial initiation, all patients admitted to the sites’ EDs will be entered in a screening log, either by part of the inclusion and randomization process or post hoc. The screening log will include patient’s ID, demographic data, and reasons for not including the patients in the trial (lack of inclusion criteria, presence of exclusion criteria, logistical reasons, in that order).

## 6.2 study sites

This multicenter study will be performed at

Bispebjerg Hospital, Bispebjerg Bakke 23, 2400 København

Amager Hvidovre Hospital, Kettegård Alle 30, 2650 Hvidovre

The site investigators, responsible for the conducting the trial specific procedures and delegation at the sites are presented in the steering group section.

## 6.3 Inclusion criteria

1. Age ≥ 18 years
2. Acute (within minutes to days) onset or worsening of subjective dyspnea
3. Oxygen saturation <92% or need of oxygen
4. At least one of the following (clinical or radiological signs of congestion):
   1. Pulmonary rales
   2. Chest X-ray or CT with pulmonary congestion
   3. Lung ultrasound with multiple B-lines

## 6.4 Exclusion criteria

1. More than 4 hours from hospital admission to randomization
2. Suspected infection or sepsis
3. Known severe COPD
4. Systolic blood pressure <90 mmHg

## 6.5 Estimated Study start and duration

We expect inclusion to begin in March 2024. Inclusion is planned to be completed after 1 year. The trial is terminated after the last patient has been assessed for 30-days. Since biochemical analyses of the research biobank samples for some of the secondary endpoints, not part of the main trial publications is expected to be performed after 30-day follow-up, the trial database and research biobank will exist in a non-anonymized format for up to 10 years following inclusion and follow-up of the last patient.

# 7. Outcomes

## 7.1 Primary outcome

## Pulmonary parenchymal fluid content measured by ReDS after 24 hours preceded by 10 minutes without oxygen-supplementation.

## 7.2 Secondary outcomes

1. Arterial blood gas concentration after 24 hours preceded by 10 minutes without oxygen-supplementation.
2. All-cause mortality at day 30
3. Days alive out-of-hospital to day 30
4. Time to freedom from oxygen-supplementation

## 7.3 Eksploratory outcomes

1. Change from baseline in log-transformed biomarkers (N-Terminal Pro-Brain Natriuretic Peptide (NTproBNP), troponin T, creatinine, neuron-specific enolase, procalcitonin) to 24 hours from admission.
2. 2D and 3D systolic and diastolic strain parameters (i.e. time to peak strain, systolic and diastolic strain rates, displacements, velocities, torsion) and traditional TTE measures as well such as ventricular volumetry and function, stroke volume derived from the velocity time integral of the left ventricular outflow tract, tissue doppler and mitral valve inflow patterns to assess diastolic function, right ventricular fractional area change and tricuspid annular plane systolic excursion.
3. Days alive out-of-ICU until day 30
4. Patient-reported dyspnea on a VAS-scale 1-100 after 24 hours
5. Number of patients where intervention is terminated (opt out) before 6 hours
6. Time within targeted SpO2-interval (89-91 % in restrictive group, 95-97% in liberal group)
7. Multiple B-lines in at least two areas on lung ultrasound identifying interstitial syndrome (yes/no).
8. FiO2, Blood pressure, respiratory rate, heart rate after 24 hours
9. Quality of life and depression: HADS, SF-36 and EQ-5D-5L

# 8. Sample size calculations and statistical analysis plan

In a validation-trial of ReDS, Amir et al investigated 16 patients with acute heart failure and found a mean fluid content of 39.8 + 6.8 % [25]. Similar results were seen when estimating fluid content by the chest CT, which is regarded as the gold standard.

To have 90 % power to find a difference of 4 % in lung fluid content (change from 39.8% to 35.8%) with a standard deviation of 6.8, we need to include 122 patients (61 in each group). The α-level is 0.05, and the beta is 10%. We expect minimal dropouts. The study will include patients over an estimated period of 1 years in two centers. We will include patients until we have reached the estimated sample size without any missing values in the primary outcome, to ensure that we can conclude on our primary hypothesis.

## 8.1 Expected enrollment

Our pilot-study of patients admitted with acute dyspnea from Bispebjerg University Hospital, we screened 707 patients during 20.5 months inclusion [29, 30]. Applying the current study criteria in that dataset, we find an incidence of eligible patients of 2-5 patients per week. We choose a conservative estimate of including approximately 1 patient per week per site. We expect to include at 2 centers, thus including expected duration of 1 year (we expect a slower inclusion in the initial phase of the study).

All included patients will continue in the study until 30 days after inclusion and/or death.

# 9. Pre-defined study-procedures

#### At inclusion and after 24 hours the patients will have a physical examination where the pulmonary respiratory sounds will be recorded as part of the evaluation of the degree of cardiac incompensation.

#### Echocardiographic parameters

At all sites, an advanced transthoracic echocardiography (TTE) will be performed at admission prior to start of intervention and again after 24 hours. Echocardiography is part of routine monitoring in these patients and is not deemed to expose the patients to excess risk. The first TTE will be performed prior to titration towards the allocated target SpO2. The primary measured outcome will be the change in longitudinal peak systolic strain (%) measured between identical TTE image acquisitions obtained in the same individual. Secondary outcome measures include the acquisition of the remaining TTE 2D and 3D systolic and diastolic strain parameters (i.e. time to peak strain, systolic and diastolic strain rates, displacements, velocities, torsion) and traditional TTE measures as well such as ventricular volumetry and function, stroke volume derived from the velocity time integral of the left ventricular outflow tract, tissue doppler and mitral valve inflow patterns to assess diastolic function, right ventricular fractional area change and tricuspid annular plane systolic excursion [31].

#### Remote Dielectric Sensing (ReDS)

Lung fluid content will be assessed noninvasively at admission and after 24 hours by the remote dielectric sensing (ReDS) device (Sensible Medical, Netanya, Israel). ReDS is an FDA-approved, wearable device developed by Sensible Medical Innovations that allows clinicians and other health care providers to quickly and accurately measure lung fluid in patients, noninvasively.

The lung fluid content is measured within the mid-region of the right lung. The measurement is presented as the percentage of the lung volume composed of fluid, (normal values for non-congested people is 20%-35% of fluid content) [25].

Using the ReDS technology in our study will seek to directly measure the degree of pulmonary congestion in acute heart failure patients and assess whether the effect of oxygen-level will be influenced by degree of pulmonary congestion at various points of care.

#### Carbon monoxide rebreathing method

The method is based on the dilution principle, where carbon monoxide binds with great affinity to circulating Hemoglobin molecules. Carbon monoxide is inhaled for 6 minutes and the increase in carboxyhemoglobin from before to after inhalation can be used to calculate the hemoglobin mass. Carboxyhemoglobin is measured at the start at time 0 and again after 8 minutes from the end of the rebreathing method (+14 min from the start). From the Hemoglobin mass, Hemoglobin and hematocrit, the total blood volume, plasma volume and erythrocyte volume can be calculated [32]. The amount of carbon monoxide that will be absorbed during the experiment is small (1.2 ml kg-1) and will only increase the level of carbon monoxide in the blood by a maximum of 12%, which is not associated with risks or discomfort [33]. The level of carbon monoxide after rebreathing is similar to the levels seen by living in a big city for 12-48 hours. Exercise capacity and circulating carbon monoxide levels in the blood return to normal levels within approx. 12 hours.

# 10. ETHICAL CONSIDERATIONS

The trial will be conducted in accordance with national legislation on medical research involving humans. The Danish legislation requires consent the patients prior to inclusion. The investigators will be responsible for application to the ethical committee and for adherence to national legislation on subject enrolment in the trial.

### 10.1 ETHICAL JUSTIFICATION

The participation and interventions in the trial will not interfere with or delay diagnostics, therapeutic procedures or medication

Potential benefits and potential harms

Interventions in this trial are routine treatments used for these patients for more than 100 years. The profile and side effects of oxygen are well-known and considered to be insignificant in the doses proposed in this trial. Details about the potential benefits of the intervention are provided in the background section.

Most importantly, if the patient is not included in this trial, the same doses of oxygen will be given to the patients in a non-systematically way as part of guideline-recommended treatment. As described in the background-section, there is clinical equipoise of which strategy that is superior in the acute setting.

Research in acute heart failure with pulmonary congestion

Research in pulmonary edema is ethically challenging for two reasons: 1) Patients can be acutely affected by dyspnea and 2) treatment must be administered within minutes. Despite these challenges, there is an ongoing need to conduct research in this patient-population to improve outcome, which is also recommended in international guidelines, such as the Declaration of Helsinki [34].

The ethical justifications for interventions in the trial are:

1. Knowledge of the optimal oxygenation therapy of patients in acute heart failure is limited and there is clinical equipoise regarding optimal oxygenation strategy.
2. The trial interventions are expected to be of minimal risk to the patient and are within current treatment guidelines, common clinical practice, and local protocols. The patients will, in other words, exclusively be presented for treatments, that are already part of the guidelines-recommended standard-of-care.
3. Increased knowledge of therapeutic potential of the intervention would increase the scientific knowledge of the condition of the individual and other patients with AHF and/or pulmonary edema, without exposing the patients to significant risk.
4. Any relevant expressed objections to participation in clinical trials of the person known to the researcher will be respected, and study participation will be terminated.
5. Inclusion in the trial may be of value to the individual patient but is valuable to the group of patients with acute heart failure in general, since further knowledge is needed to continue optimization of the interventions for this condition.

## 9.2 Informed consent

The trial-protocol will be sent for approval by the ethics committee.

Consent will be obtained from the patient when he/she is deemed capable for the information and will be asked for a written informed consent (paper or in REDcap).

At any time, if the patient asks to be taken out of the trial, this will be respected. If a patient denies future participation in the trial, no additional data will be collected but all data collected up until the point of withdrawal will be included consistent with Danish law.

Information

Informed consent will include information that the hospital uses the best evidence-based therapy to relieve dyspnea for this patient. The patient is told that two equal treatment options exist, and we will give one of them after randomization, provided there are no contraindications or exclusion criteria. On the possible inclusion in the trial, every measure possible will be taken to ensure a quiet environment for the information.

Insurance

The patients in the study are covered by the Danish patient insurance.

# 11. Monitoring

### 11.1 Good Clinical Practice monitoring

The trial will not be monitored for Good Clinical Practice, since this is not required in non-drug trials. However, the GCP standards will be followed.

## 11.2 Access to Source Data and Documentation

The principal investigator will permit monitoring, audits, reviews of ethical committees and regulatory authorities’ direct access to source data, blinded to treatment allocation. Informed consent includes an accept of the data access in the study’s eCRF for the principal investigator and further the Danish Medicines Agency for quality control. Information on written consent for both patients and nearest relatives includes information on data access.

# 12. Statistical analyses plan

Baseline characteristics and outcomes of the patients in the groups will be compared individually. The primary exposure variable is allocation to the intervention group compared to control group. Categorical variables will be presented as numbers (frequencies) and compared with the Chi-Squared or the Fisher’s exact test, as appropriate, whereas continuous variables will be presented as mean ±SD if normally distributed, and as median (25^th^ percentile-75^th^ percentile) if non-normally distributed and compared with the T-test. In continues data, there are non-normally distributed, logarithmic transformation will be applied to approximate normal distribution as appropriate. Repeated measurements, mixed models will be made for analysis of the continuous variables with multiple measurements.

Missing data

In case of missingness greater than 5% for the primary endpoint, multiple imputations by chained equations will be applied as sensitivity analysis with generation of 10 individual data sets.

For other endpoints, first a worst case and best-case analysis will be made and if significant differences, multiple imputations will be performed.

Analysis of survival data and other outcomes

Kaplan-Meier curves for each group will be graphically displayed and compared by the log-rank test. Further multivariable Cox proportional hazard models will be applied to assess differences in time to death between treatment groups. These models will sequentially be adjusted for the interaction between treatment allocation, and each of the predefined covariates. Further, models stratified by individual components of the predefined covariates will be made as hypothesis generating. Any changes from the pre-specified analysis plan will be reported.

Outcomes will be evaluated using univariable and multivariable logistic regression models after examining assumptions of linearity and proportionality. Hazard ratios (HRs) and Odds ratios (ORs) with corresponding two-sided 95% confidence intervals (CI) will be presented.

Multivariable models are adjusted for:

*Admission SBP (quartiles), age, sex, body mass index, LVEF at admission (>50, 40-50, <40), NT-proBNP (above/below median), eGFR, supraventricular arrhythmia at admission, comorbidities (history of heart failure, obstructive pulmonary disease), systemic congested (Yes/no, defined as at least 3 kg above ideal weight at admission).*

The covariates are pre specified before analyses and were chosen based on a consensus of the steering committee based on experiences from previous trials of AHF. Interaction between covariates will be tested by adding the interaction term to the models.

Subgroup analysis

Subgroup analysis will be made for all the prespecified covariates. Interaction with treatment effect will be tested for each covariate and a forest plot will be plotted.

Two-sided p-value of less than 0.05 is considered statistically significant. All analyses will be conducted in the modified intention-to-treat (ITT) (all randomized patients fulfilling the inclusion criteria and excluding those, which consent have been withdrawn) population. Statistical analyses are performed using the SAS statistical software, version 9.4 (SAS Institute, Cary, NC) and in R version 3.3.3 (R Foundation for Statistical Computing, Vienna, Austria).

# 12. Data collection and management

The trial database/eCRF will be constructed using REDCap software, hosted by the Capital Region. This database complies with Danish data safety legislation.

The data protection act and the data protection regulation is complied with regarding collection and storage of biological material and journal data. No material will be sent abroad.

A research biobank will be established. We will draw blood samples during hospitalizations at admission and after 24 hours. These blood samples will be stored for later analysis (research biobank, approx. 50 ml in total). The purpose of the research biobank is to investigate the effect of oxygen on biomarkers for heart, brain, kidney function and inflammatory reaction. The material is stored for up to 10 years, after which it is destroyed, no later than 1 February 2036. The material is stored pseudo-anonymized. Material will not be sent abroad.

## 11.1 Data sources

Data capture for the eCRF originates from the following sources:

- The screening and randomization log (REDCap survey)
- The eCRF, to be filled out by the site investigator or delegate (Patient electronic chart to CRF)
- Follow-up mailed questionnaires (REDCap surveys)
- Paper consent form or consent from REDcap-survey. Scanned and filed in REDCap AND stored in paper format at the site.
- Biochemistry data pull from LABKA
- Research biobank analyses in Excel, patient trial ID as identifier
- Electronic medical record
- Data from medical devices: Echo-machines, ReDS-machines, and O2MATIC-machines.

## 11.2 Data collection process

In the paper inlay in the sealed box the QR-code (link) to the screening survey in REDcap to fill out at inclusion. In the screening survey, the investigator will enter:

#### Screening

- Fulfilling inclusion/exclusion criteria (yes/no),
- Patient ID (CPR-number),
- running number of the study-medicine kit,
- consent (yes/no) and name from legal guardian
- If the investigator finds the patient eligible for inclusion in the trial (yes/no)

#### Medical history characteristics (at the time of inclusion, T0)

- Age
- Sex
- Height in cm
- Weight in kg (usual weight)
- Previous New York Heart Association (NYHA) classification
- Comorbidity (Heart failure, AMI, arrhythmia, arterial hypertension, previous stroke, previous transitory cerebral ischemia, diabetes type I, diabetes type II, asthma or COPD, renal failure, liver cirrhosis, malignancy, alcohol abuse, drug abuse, HIV, AIDS, previous PCI, previous CABG, previous valve surgery)
- Medications
- Pervious LVEF
- Inclusion / exclusion criteria

#### Data from emergency department (at the time of inclusion, T0)

- Weight in kg (measured in the ED)
- Time of symptom-onset
- Administered study-medicine
- Administered treatment prehospital
- Time of hospital arrival
- Time from hospital arrival to inclusion (calculated)
- Including site
- Blood gas analyses
- Echocardiography if available
- Vital signs at admission and after 1- and 6-hours including blood pressure, heart rate, respiratory rate, and peripheral oxygen saturation.
- Any concomitant therapy
- Routine biochemistry**
- ECG findings (Rhythm; signs of ischemia and QRS configuration: normal, STEMI, NSTEMI, LBBB, RBBB, other)

#### Characteristics Daily During hospitalization

- Echocardiography
- Vital signs
- Any concomitant therapy
- Heart failure medicine
- Biochemistry**
- ECG findings (Rhythm; signs of ischemia and QRS configuration: normal, STEMI, NSTEMI, LBBB, RBBB, other)
- Need for pacing (yes/no)
- Need for Angiography (yes/no)
- Time to PCI (yes/no)
- Time to CABG (yes/no)
- Need for ICU (yes/no)
- Need for vasopressors or inotropes (yes/no)
- Need for mechanical ventilation (yes/no)
- Need for renal replacement
- Chest x-ray (yes/no)
- CT for pulmonary embolism (yes/no)
- Telemetry
- Cardiac arrest
- cardiogenic shock (yes/no)
- urine production

**Routine biochemistry includes: Troponin T, CKMB, NT-pro-BNP, creatinine, ferritin, neuron Specific Enolase, CRP, procalcitonin, leukocyte- and differential count, albumin, ALAT, ASAT, bilirubin, INR, creatinine, sodium, potassium, calcium, glucose, albumin, blood urea nitrogen, uric acid, red blood cell count, platelet count, HbA1c, TSH, transferrin, ferritin.

*Dead*:

- Y/N

If dead, specify presumed cause:

- Cardiovascular: progressive cardiogenic shock or fatal arrhythmia, heart failure
- Cerebral: cerebral herniation and/or severe intracranial bleeding, or withdrawal of life sustaining therapies due to brain injury
- Sepsis
- Multi Organ Dysfunction Syndrome (MODS)

*Hospital discharge*

- Time and results of MRI, CT, Echocardiography, Angiography
- PCI performed
- Number of coronary interventions
- CABG performed
- ICD inserted
- GCS at discharge
- mRS at discharge
- Length of initial hospital stay (counted from admission till discharge to home, rehabilitation facility, or death)
- LVEF at discharge/last recorded during initial hospital stay (for select centers also ECHO on day 1 and 3-5)
- Discharged to ward/home/nursing home/rehabilitation unit
- Dead before discharge

Details, if applicable:

- Echocardiography
- Pacing
- ICU
- vasopressors or inotropes
- mechanical ventilation
- renal replacement
- Chest x-ray
- CT for pulmonary embolism

#### Follow-up assessments

Follow-up assessments will occur after 30-day post-inclusion. The 30-day assessment will be done over telephone and by present visit on the hospital. The following will be recorded:

- The occurrence of any specific adverse events
- The occurrence of events defined as endpoints
- Days alive out of hospital at 30 days.
- Survival: Y/N
- Additional data capture of above listed items related to but not recorded at previous visits/initial hospital stay
- Days out of ICU and out of hospital during the first 30 days

#### Long-term follow-up

Registry based follow-up after 5 and 10 years.

- The occurrence of events defined as endpoints
- Survival: Y/N

# 13. Clinical treatment outside the treatment allocation

Besides the intervention, all patients will be treated according to current regional, national, and international guidelines at the discretion of the treating physician. Long term heart failure treatment will be planned by the treating physician according to patient preferences.

# 14. ADVERSE EVENT REPORTING

Patients in pulmonary edema are always acutely and sometimes chronic sick with a high morbidity and mortality (please see Background). Accordingly, it is difficult or impossible to assess and report all adverse events and the potential relationship with any given intervention. The overall benefit versus harm of the interventions will be assessed and reported through the primary and secondary outcomes of the trial. Accordingly, events that are frequently occurring during initial hospital-stay for these patients will therefore NOT be considered an AE in the present study include the following:

- Minor bleeding: A bleeding episode that cannot be categorized as “major” (see below) from nose, gastro-intestinal tract, oral cavity, insertion sites, intramuscular, or another site.
- Metabolic disorder: Intermittent hypo- or hyperglycemia.
- Other biochemical abnormalities: AHF-patients will frequently have highly elevated levels of acute phase reactants including CRP and fibrinogen during initial ICU stay, as well as elevated creatinine, and other biochemical markers of organ injury. Only if such is considered deviating from what can be expected in this category of patients will it be recorded as an AE/AR.

Patients will be monitored for the occurrence of an adverse event for 30 days after randomization or until hospital discharge if this occurs later than 30 days and will not be followed for adverse event reporting after this. Adverse events that in general will be potentially related to the study interventions will include death, hypotension, headache, hypo- or hyperkalemia, hyponatremia, fluid retention (peripheral edema), loss of hearing and anaphylaxis.

## 14.1 Definitions

All applied definitions originate from the EU regulation on clinical trials on medicinal products for human use, and repealing Directive 2001/20/EC.

### 14.1.1 Adverse events

An adverse event means any untoward medical occurrence in a subject to whom a medicinal product is administered, and which does not necessarily have a causal relationship with this treatment.

Adverse events (AE) will be categorized according to the definitions below. To assess specific adverse events possibly related to the trial intervention, we will collect data on the following during the first 30 days after inclusion in the trial:

- Symptomatic hypotension needing medical therapy.
- Major bleeding: Bleeding causing fatality, symptomatic intracranial bleeding.
- Renal impairment: Need for continuous renal replacement therapy or intermittent hemodialysis.
- Cardiac arrhythmias: VF, VT and AF requiring DC conversion, new need for pacing during the first 24 hours.
- Respiratory: Intubation and mechanical ventilation during admission.
- Other adverse events potentially related to the intervention with specification.

No other events will be defining as AEs.

### 14.1.2 Serious adverse events

A serious adverse event is defined by regulatory agencies as one that suggests a significant hazard or side effect, regardless of the investigator’s or sponsor’s opinion on its relationship to investigational product. This includes, but may not be limited to, any event that (at any dose):

- is fatal
- is life-threatening (places the subject at immediate risk of death)
- requires in-patient hospitalization or prolongation of existing hospitalization
- is a persistent or significant disability/incapacity
- results in a congenital anomaly or birth defect

A hospitalization meeting the regulatory requirement for “serious” criteria is any in-patient hospital admission or prolongation hereof should be considered a serious adverse event.

Any event that does not exactly meet this definition yet which, in the investigator’s opinion, represents a significant hazard can be assigned the “other significant hazard” regulatory reporting serious criteria.

Additionally, important medical events that may not be immediately life threatening or result in death or hospitalization, but which may jeopardize a subject or require intervention to prevent one of the outcomes listed above, or result in urgent investigation, may be considered serious. Examples include allergic bronchospasm, convulsions, and blood dyscrasias.

Severity of adverse events

For each AE, severity will be graded accordingly:

- Mild: Transient symptoms, no interference with patient’s daily activities.
- Moderate: Marked symptoms, moderate interference with patient’s daily activities.
- Severe: Considerable interference with patient’s daily activities.

Relationship of AE to trial intervention

For each AE, relationship to trial intervention will be rated accordingly:

- Probable: Good reason and enough documentation to assume a causal relationship.
- Possible: A causal relationship is likely and cannot be excluded.
- Unlikely: The event is most likely related to an etiology other than the intervention.
- Unknown: Causality is not assessable.

An AE with a possible or probable relationship to the trial intervention will be categorized as an adverse reaction (AR).

## 13.5 Reporting

At each assessment of an AE/AR, it must be evaluated if it should be categorized as a serious adverse event/ (SAE), and if so, this must be reported to the trial sponsor. The trial sponsor will evaluate all SAE/SAR.

Death resulting from circulatory collapse will be recorded but will not be reported to the ethical committee outside the annual reports, as AHF patients have a ≥20% risk of death within 30 days. The death rate will have no essential information in the monitoring of trial by authorities.

All SAEs occurring at the trial centers will be submitted once a year by the sponsor and a safety report of all trial patients will be submitted to the ethical committee.

No later than 90 days after the trial has been completed, the trial sponsor will notify the ethical committee, and if the trial is stopped earlier than planned, the reasons will be reported.

## 13.6 Reporting Procedures for All Adverse Events

All adverse events occurring after inclusion observed by the investigator or reported by the subject (whether attributed to investigational product), will be reported on the case report form. Medically significant adverse events considered related to the investigational product by the investigator or the sponsor will be followed until resolved or considered stable.

The following attributes must be assigned by the investigator: description; dates of onset and resolution; severity; assessment of relatedness to investigational product, other suspect drug, or device, and action taken. The investigator may be asked to provide follow-up information.

It will be left to an investigator’s clinical judgment as to whether an adverse event is of sufficient severity to require a subject’s removal from treatment. A subject may also withdraw voluntarily from treatment because of what he or she perceives as an intolerable adverse event. If either of these situations arises, the subject must undergo an end-of-study assessment and be given appropriate care under medical supervision until symptoms cease or the condition becomes stable. If the subject was permanently withdrawn from the study or investigational product due to a serious adverse event, this information must be included in either the initial or follow-up Serious Adverse Event Report Form or the End of Study Case Report Form. Data on deaths and hospitalizations will be collected until study termination.

# 14. Timeline and enrollment

We expect to start inclusion per 1. May 2023

|  | **Pre-trial** | **Year 1** | | | | **Year 2** | | | | **Year 3** | | | |
| --- | --- | --- | --- | --- | --- | --- | --- | --- | --- | --- | --- | --- | --- |
| **Funding** |  |  |  |  |  |  |  |  |  |  |  |  |  |
| **Protocol development and modifications** |  |  |  |  |  |  |  |  |  |  |  |  |  |
| **Ethical approval** |  |  |  |  |  |  |  |  |  |  |  |  |  |
| **Creation of database** |  |  |  |  |  |  |  |  |  |  |  |  |  |
| **Trial registration** |  |  |  |  |  |  |  |  |  |  |  |  |  |
| **Creation of randomization list** |  |  |  |  |  |  |  |  |  |  |  |  |  |
| **Education of site personnel** |  |  |  |  |  |  |  |  |  |  |  |  |  |
| **Enrollment and assessment of outcomes** |  |  |  |  |  |  |  |  |  |  |  |  |  |
| **Writing and publication of Design article** |  |  |  |  |  |  |  |  |  |  |  |  |  |
| **Cleaning and closing of the database** |  |  |  |  |  |  |  |  |  |  |  |  |  |
| **Data analyses** |  |  |  |  |  |  |  |  |  |  |  |  |  |
| **Main manuscript-writing** |  |  |  |  |  |  |  |  |  |  |  |  |  |
| **Unblinding** |  |  |  |  |  |  |  |  |  |  |  |  |  |
| **Publication and presentation of results** |  |  |  |  |  |  |  |  |  |  |  |  |  |

# 15. Funding

The steering group has initiated the study. The trial will be funded by external foundations for medical research. The trial is an investigator-initiated trial, and the budget will be covering expected expenses only. Gangstedfonden has supported the project with 450.000 kr. When additional funding is received the ethics committee will be noticed.

# 16. Publication plan

All trial analyses will be conducted by the steering committee, and the results will be interpreted with treatment allocation coded as “1” and “2”. After acceptance of the analysis and conclusions by the steering committee, the trial will be unblinded.

## 16.1 Design paper

A design paper describing the study design, trial population definition including inclusion and exclusions criteria, endpoints as well as the planned statistical analyses plan will be submitted for peer review and publication well before the main trial data analyses will begin.

Authors of the Design paper will be IA (first author), Site Investigators, study statistician and steering group member. Additional authors may be included if the Steering Group allow it and provided, and they fulfil the Vancouver criteria.

## 16.2 Main results paper

Authorship will be granted according to ICMJE guidelines. The final manuscripts of the interventions will be submitted as 1 manuscript to a peer-reviewed international journal and the findings of the study will be published regardless the results suggesting a beneficial effect, neutral effect, or harm of the interventions. The main trial paper manuscript will be drafted according to the statistical analysis plan by the Steering group members. Authors of the main trial manuscript are all, who have contributed to the trail sufficiently to be a coauthor. The expected number of coauthors will be 5 members per site and additionally 1 person per 100 included patients, in addition to the following:

IA (first author), Steering group members, study statistician, primary investigator. The target journal is a high-ranking international medical journal.

The main trial results will be published regardless of being positive, neutral, or negative. The main trials will also be submitted for presentation at national and international conferences

## 16.3 Auxiliary manuscripts / post-hoc analyses

The steering group will manage post-hoc sub-study analysis and requests for sub-study proposals before the start of main trial data analysis. The purpose of the sub-study group is to ensure an even access to the sub-studies among the participating sites and others, to avoid overlap in published sub-studies and to oversee that sub-studies are progressing according to the plan.

# 17. Data sharing

The database will be maintained for 15 years and anonymized if requested by relevant authorities. There are currently no plans of data sharing. Research biobank data, secondary analyses of imaging materials or the main trial database for sub-studies will not be made publicly available.

# 18. Tasks and responsibilities

## 18.1 Sponsor/PI

Principle Investigator (JG): Sponsor, Coordination of protocol development, funding, ethical approval, information to sites, recruitment of trial sites, daily management, authorize invoices.

Senior Investigator (JH): Funding, information, recruitment of trial sites.

## 18.2 Steering group

Protocol development, funding, information to study participant and clinical personnel, recruitment of trial sites, sub-study coordination.

Additional sites

In case target enrolments are not met after 6 months of enrolment, additional regions of Denmark will be approached regarding participation in the trial.

# 19. References

1. CH Expert Working Group ICH HARMONISED TRIPARTITE GUIDELINE GENERAL CONSIDERATIONS FOR CLINICAL TRIALS

2. Chan A-W, Tetzlaff JM, Altman DG, et al (2015) SPIRIT 2013 Statement: defining standard protocol items for clinical trials. Rev Panam Salud Publica Pan Am J Public Health 38:506–514

3. Virani SS, Alonso A, Aparicio HJ, et al (2021) Heart Disease and Stroke Statistics-2021 Update: A Report From the American Heart Association. Circulation 143:e254–e743. https://doi.org/10.1161/CIR.0000000000000950

4. Storrow AB, Jenkins CA, Self WH, et al (2014) The Burden of Acute Heart Failure on U.S. Emergency Departments. JACC Heart Fail 2:269–277. https://doi.org/10.1016/j.jchf.2014.01.006

5. Jencks SF, Williams MV, Coleman EA (2009) Rehospitalizations among patients in the Medicare fee-for-service program. N Engl J Med 360:1418–1428. https://doi.org/10.1056/NEJMsa0803563

6. Heidenreich PA, Trogdon JG, Khavjou OA, et al (2011) Forecasting the future of cardiovascular disease in the United States: a policy statement from the American Heart Association. Circulation 123:933–944. https://doi.org/10.1161/CIR.0b013e31820a55f5

7. Gheorghiade M, Abraham WT, Albert NM, et al (2006) Systolic blood pressure at admission, clinical characteristics, and outcomes in patients hospitalized with acute heart failure. JAMA 296:2217–2226. https://doi.org/10.1001/jama.296.18.2217

8. Bueno H, Ross JS, Wang Y, et al (2010) Trends in length of stay and short-term outcomes among Medicare patients hospitalized for heart failure, 1993-2006. JAMA 303:2141–2147. https://doi.org/10.1001/jama.2010.748

9. Crespo-Leiro MG, Anker SD, Maggioni AP, et al (2016) European Society of Cardiology Heart Failure Long-Term Registry (ESC-HF-LT): 1-year follow-up outcomes and differences across regions. Eur J Heart Fail 18:613–625. https://doi.org/10.1002/ejhf.566

10. Butt JH, Fosbøl EL, Gerds TA, et al (2020) Readmission and death in patients admitted with new-onset versus worsening of chronic heart failure: insights from a nationwide cohort. Eur J Heart Fail 22:1777–1785. https://doi.org/10.1002/ejhf.1800

11. Pang PS, Givertz MM (2013) The challenge of drug development in acute heart failure: balancing mechanisms, targeting patients, and gambling on outcomes. JACC Heart Fail 1:442–444. https://doi.org/10.1016/j.jchf.2013.08.004

12. McDonagh TA, Metra M, Adamo M, et al (2021) 2021 ESC Guidelines for the diagnosis and treatment of acute and chronic heart failure. Eur Heart J ehab368. https://doi.org/10.1093/eurheartj/ehab368

13. Pang PS, Collins SP, Miró Ò, et al (2017) Editor’s Choice-The role of the emergency department in the management of acute heart failure: An international perspective on education and research. Eur Heart J Acute Cardiovasc Care 6:421–429. https://doi.org/10.1177/2048872615600096

14. Javaloyes P, Miró Ò, Gil V, et al (2019) Clinical phenotypes of acute heart failure based on signs and symptoms of perfusion and congestion at emergency department presentation and their relationship with patient management and outcomes. Eur J Heart Fail 21:1353–1365. https://doi.org/10.1002/ejhf.1502

15. Chioncel O, Mebazaa A, Harjola V-P, et al (2017) Clinical phenotypes and outcome of patients hospitalized for acute heart failure: the ESC Heart Failure Long-Term Registry. Eur J Heart Fail 19:1242–1254. https://doi.org/10.1002/ejhf.890

16. Ponikowski P, Voors AA, Anker SD, et al (2016) 2016 ESC Guidelines for the diagnosis and treatment of acute and chronic heart failure: The Task Force for the diagnosis and treatment of acute and chronic heart failure of the European Society of Cardiology (ESC)Developed with the special contribution of the Heart Failure Association (HFA) of the ESC. Eur Heart J 37:2129–2200. https://doi.org/10.1093/eurheartj/ehw128

17. Sepehrvand N, Ezekowitz JA (2016) Oxygen Therapy in Patients With Acute Heart Failure: Friend or Foe? JACC Heart Fail 4:783–790. https://doi.org/10.1016/j.jchf.2016.03.026

18. Park JH, Balmain S, Berry C, et al (2010) Potentially detrimental cardiovascular effects of oxygen in patients with chronic left ventricular systolic dysfunction. Heart Br Card Soc 96:533–538. https://doi.org/10.1136/hrt.2009.175257

19. Farquhar H, Weatherall M, Wijesinghe M, et al (2009) Systematic review of studies of the effect of hyperoxia on coronary blood flow. Am Heart J 158:371–377. https://doi.org/10.1016/j.ahj.2009.05.037

20. Haque WA, Boehmer J, Clemson BS, et al (1996) Hemodynamic effects of supplemental oxygen administration in congestive heart failure. J Am Coll Cardiol 27:353–357. https://doi.org/10.1016/0735-1097(95)00474-2

21. Mak S, Azevedo ER, Liu PP, Newton GE (2001) Effect of hyperoxia on left ventricular function and filling pressures in patients with and without congestive heart failure. Chest 120:467–473. https://doi.org/10.1378/chest.120.2.467

22. Schmidt H, Kjaergaard J, Hassager C, et al (2022) Oxygen Targets in Comatose Survivors of Cardiac Arrest. N Engl J Med. https://doi.org/10.1056/NEJMoa2208686

23. Hofmann R, James SK, Jernberg T, et al (2017) Oxygen Therapy in Suspected Acute Myocardial Infarction. N Engl J Med 377:1240–1249. https://doi.org/10.1056/NEJMoa1706222

24. Sepehrvand N, Alemayehu W, Rowe BH, et al (2019) High vs. low oxygen therapy in patients with acute heart failure: HiLo-HF pilot trial. ESC Heart Fail 6:667–677. https://doi.org/10.1002/ehf2.12448

25. Amir O, Azzam ZS, Gaspar T, et al (2016) Validation of remote dielectric sensing (ReDS^TM^) technology for quantification of lung fluid status: Comparison to high resolution chest computed tomography in patients with and without acute heart failure. Int J Cardiol 221:841–846. https://doi.org/10.1016/j.ijcard.2016.06.323

26. Amir O, Rappaport D, Zafrir B, Abraham WT (2013) A novel approach to monitoring pulmonary congestion in heart failure: initial animal and clinical experiences using remote dielectric sensing technology. Congest Heart Fail Greenwich Conn 19:149–155. https://doi.org/10.1111/chf.12021

27. Ezekowitz JA, Hernandez AF, O’Connor CM, et al (2012) Assessment of dyspnea in acute decompensated heart failure: insights from ASCEND-HF (Acute Study of Clinical Effectiveness of Nesiritide in Decompensated Heart Failure) on the contributions of peak expiratory flow. J Am Coll Cardiol 59:1441–1448. https://doi.org/10.1016/j.jacc.2011.11.061

28. Hansen EF, Hove JD, Bech CS, et al (2018) Automated oxygen control with O2matic® during admission with exacerbation of COPD. Int J Chron Obstruct Pulmon Dis 13:3997–4003. https://doi.org/10.2147/COPD.S183762

29. Nielsen OW, Valeur N, Sajadieh A, et al (2019) Echocardiographic subtypes of heart failure in consecutive hospitalised patients with dyspnoea. Open Heart 6:e000928. https://doi.org/10.1136/openhrt-2018-000928

30. Miger KC, Fabricius-Bjerre A, Maschmann CP, et al (2021) Clinical Applicability of Lung Ultrasound Methods in the Emergency Department to Detect Pulmonary Congestion on Computed Tomography. Ultraschall Med Stuttg Ger 1980 42:e56. https://doi.org/10.1055/a-1065-7409

31. Fischer K, Ranjan R, Friess J-O, et al (2021) Study design for a randomized crossover study investigating myocardial strain analysis in patients with coronary artery disease at hyperoxia and normoxemia prior to coronary artery bypass graft surgery (StrECHO-O2). Contemp Clin Trials 110:106567. https://doi.org/10.1016/j.cct.2021.106567

32. Bomholt T, Larsson S, Rix M, et al (2020) Intravascular volumes evaluated by a carbon monoxide rebreathing method in patients undergoing chronic hemodialysis. Hemodial Int 24:252–260. https://doi.org/10.1111/hdi.12820

33. Rose JJ, Wang L, Xu Q, et al (2017) Carbon Monoxide Poisoning: Pathogenesis, Management, and Future Directions of Therapy. Am J Respir Crit Care Med 195:596–606. https://doi.org/10.1164/rccm.201606-1275CI

34. World Medical Association (2013) World Medical Association Declaration of Helsinki: ethical principles for medical research involving human subjects. JAMA 310:2191–2194. https://doi.org/10.1001/jama.2013.281053

# Appendix 1: Steering group conflicts of interest

Johannes Grand

- Industry: none
- Other: None
- Date of up-date: 31-07-2022

Jens Hove

- Industry: none
- Other: is co-owner of the company O2matic Aps, but does not receive any fees or project support from O2matic in this connection.
- Date of up-date: 31-07-2022

Christian Hassager

- Industry: none
- Other: None
- Date of up-date: 31-07-2022

Ejvind Frausing

- Industry: none
- Other: is co-owner of the company O2matic Aps, but does not receive any fees or project support from O2matic in this connection.
- Date of up-date: 31-07-2022

Olav Wendelboe nielsen

- Industry: none
- Other: None
- Date of up-date: 31-07-2022

Ida Arentz Taraldsen

- Industry: none
- Other: None
- Date of up-date: 31-07-2022

# Appendix 2: Generic flow chart containing information on when and how to include patients

1. Check inclusion / exclusion criteria (on pocked card) to ensure eligibility.
2. Informed consent.
3. Scan QR-code on a *REDOX pocket card* to open REDcap-screening survey on smartphone or tablet (alternative a link on the pocked card can be entered in a PC-browser).
4. Enter screening information as requested and randomize in REDcap.
5. Perform study related procedures.
6. Set-up the O2MATIC and adjust to allocated target.
7. Put O2MATIC on black-screen to ensure blinding.

**Informeret samtykke til deltagelse i et sundhedsvidenskabeligt forskningsprojekt**

Optimal iltbehandling ved åndenød og væske i lungerne

Original forsøgstitel: RESTRICTIVE VERSUS LIBERAL OXYGENATION TARGETS IN PATIENTS WITH ACUTE HEART FAILURE – A RANDOMIZED CLINICAL TRIAL

**Projektet er godkendt af den Videnskabsetiske komite.**

**Erklæring fra forsøgspersonen:**

Jeg har fået skriftlig og mundtlig information og jeg ved nok om formål, metode, fordele og ulemper til at give mit samtykke. Jeg ved, at det er frivilligt at deltage, og at jeg altid kan trække mit samtykke tilbage uden at miste mine nuværende eller fremtidige rettigheder til behandling.

Jeg giver samtykke til, at jeg deltager i forskningsprojektet og til at mit biologiske materiale udtages med henblik på opbevaring i en forskningsbiobank.

Forsøgspersonens navn: ________________________________________________________

Dato: _______________ Underskrift: ____________________________________________

Hvis der kommer nye væsentlige helbredsoplysninger frem om Dem i forskningsprojektet vil De blive informeret. Vil De **frabede** dig information om nye væsentlige helbredsoplysninger, som kommer frem i forskningsprojektet, bedes De markere her: __________ (sæt x). Hvis De vil **frabede** dig at blive informeret om forskningsprojektets resultat når projektet er slut, bedes De sætte kryds her: Nej _____ (sæt x)

**Erklæring fra den, der afgiver information:**

Jeg erklærer, at der er afgivet mundtlig og skriftlig information om forsøget.

Efter min overbevisning er der givet tilstrækkelig information til, at der kan træffes beslutning om forsøgspersonens deltagelse i forsøget.

Navnet på den, der har afgivet information:

Dato: _______________ Underskrift: ______________________________________________

Dato og underskrift fra patient og informerende læge laves elektronisk identisk med denne formular i REDCap og kobles til patientens forløb.

# Appendix 4: study-related procedures

| Time | **Screening, baseline pre-randomization assessment** | **Randomization** | **12 h post randomization** | **24 h post randomization** | **Discharge** |
| --- | --- | --- | --- | --- | --- |
| Informed consent, patient | x |  |  |  |  |
| Demographics | x |  |  |  |  |
| Inclusion/ exclusion criteria | x |  |  |  |  |
| Medical history | x |  |  |  |  |
| Concomitant medications | x |  |  | x | x |
| Routine blood test | x |  |  | x (morning) |  |
| Arterial blood gas | x |  | x | x |  |
| ECG | x |  |  | x |  |
| Vital signs | x |  | x | x |  |
| Chest x-ray | x |  |  |  |  |
| Echocardiography | x |  |  | x |  |
| Focus lung-ultrasound | X |  |  | x |  |
| ReDS | X |  |  | X |  |
| Patient-dyspnea | X |  |  | X |  |
| Plasma volume | X |  |  | X |  |
| 5Q-DL |  |  |  |  | x |
| Adverse events reporting |  |  |  |  | x |

# Appendix 5: Timeline


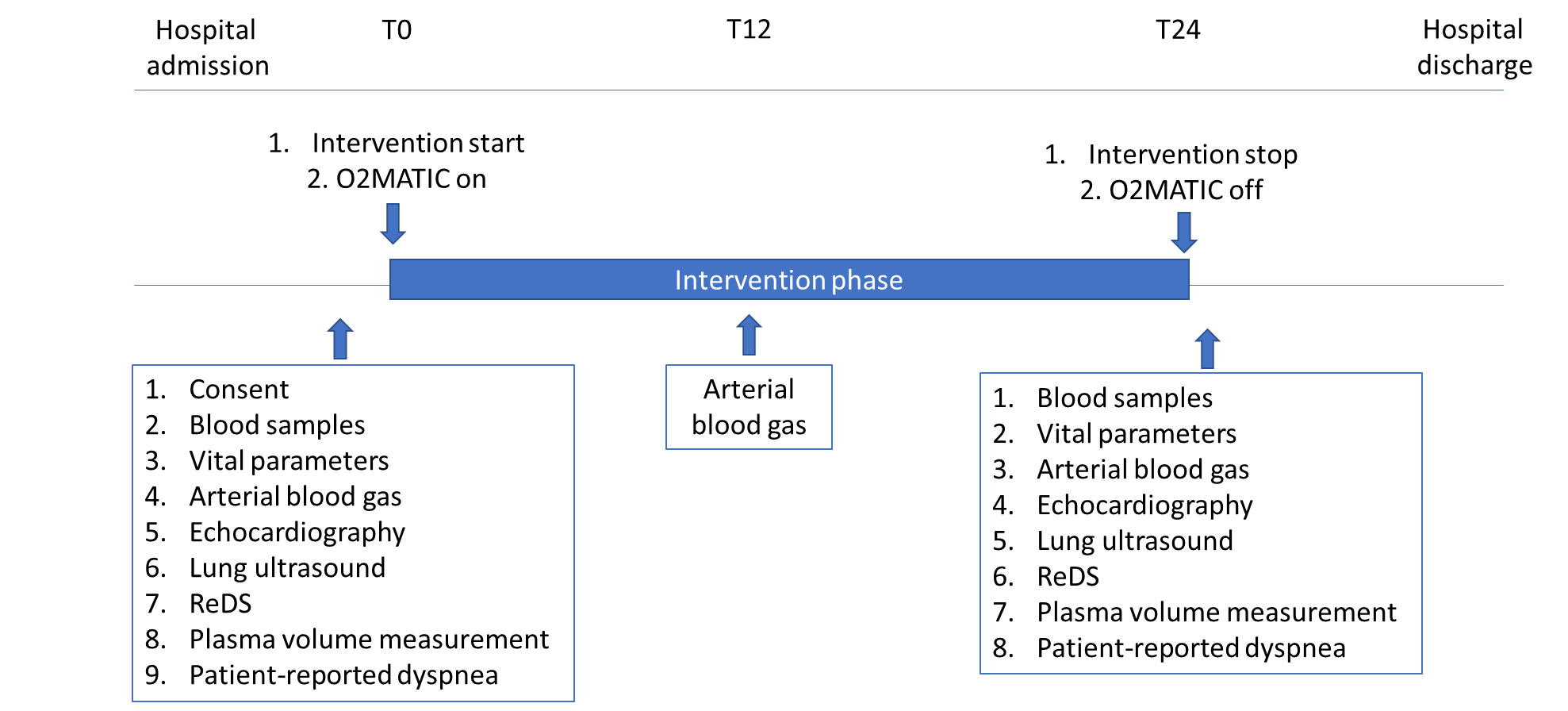


# Appendix 6: Acronyms and abbreviations

ACC/AHA American College of Cardiology/American Heart Assocation

AE Adverse events

ACE Angiotensin-converting enzyme

ACS Acute Coronary Syndrome

ADHF/AHF Acute decompensated heart failure/Acute heart failure

AF Atrial fibrillation

AKI Acute kidney injury

ARB Angiotensin II receptor blockers

BB Beta-blocker

BMI Body Mass Index

BNP B-type natriuretic peptide

BP Blood pressure

CAD Coronary artery disease

CABG Coronary artery bypass grafting

CHF Chronic heart failure

CI Confidence intervals

CKD Chronic kidney disease

COPD Chronic obstructive pulmonary disease

CRT Cardiac resynchronisation therapy

CXR Chest X-ray

EF Ejection fraction

EGFR Estimated glomerular filtration rate

ELISA Enzyme-linked immunosorbent assay

ESC European Society of Cardiology

FVC Forced vital capacity

HFPEF Heart failure with preserved ejection fraction

HR Hazard ratio

HRQoL Heath-related quality of life

ICD Implantable Cardioverter Defibrillators

ICU Intensive care unit

IHD Ischemic heart disease

INR International normalized ratio

IQR Interquartile range

ITT Intention-to-treat analysis

IV Intravenous

JVP Jugular venous pressure

LVAD Left Ventricular Assist Device

LVEF Left Ventricular Ejection Fraction

MI Myocardial infarction

Minnesota LWHFQ Minnesota Living With Heart Failure Questionnaire

MRA Mineralocorticoid receptor antagonists

NSAIDS Non-steroidal anti-inflammatory drugs

NT-proBNP N-terminus pro B type natriuretic peptide

NYHA New York Heart Association Classification

PCI Percutaneous coronary intervention

PICO Population, intervention, comparison and outcome

QALY Quality-adjusted life years

QOL Quality of life

RAS Renin-angiotensin-system

RCT Random control trial

ROC Receiver operating characteristics

SBP Systolic Blood Pressure

SD Standard deviation

SE Standard error

TIA Transient ischaemic attack

VAS Visual analogue scale
