# Supplementary figures and images for: Restrictive versus liberal oxygenation targets in patients with acute heart failure and pulmonary congestion–A protocol for a Randomized Controlled Trial (The REDOX-AHF trial)
Source: PLoS One. 2026 May 22;21(5):e0349791. doi: 10.1371/journal.pone.0349791 (PMC13196945; doi:10.1371/journal.pone.0349791)

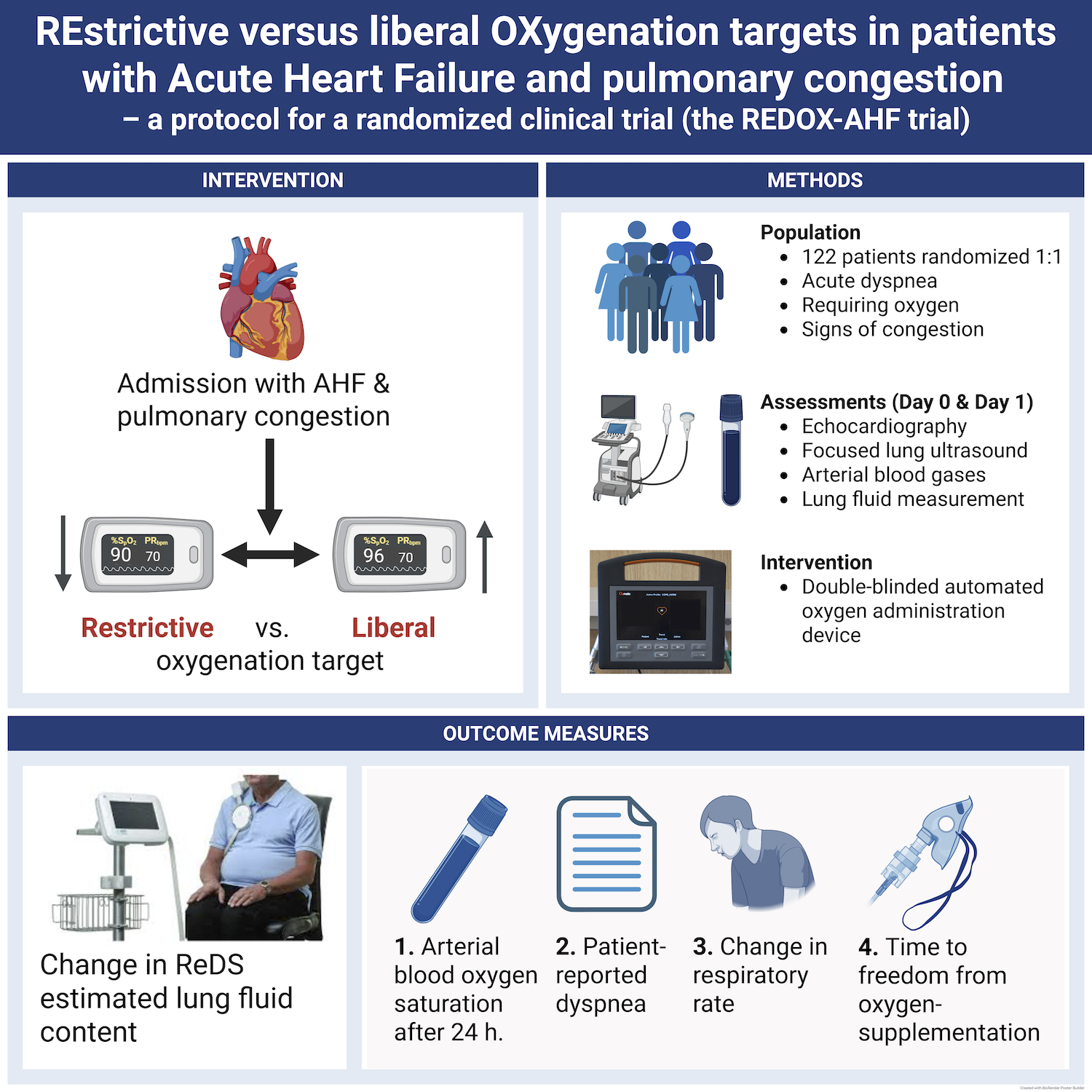

Supplement: S1 Fig — (TIFF) [file pone.0349791.s004.tiff]
